# Effect of an analgo-sedation protocol for neurointensive patients: a two-phase interventional non-randomized pilot study

**DOI:** 10.1186/cc8978

**Published:** 2010-04-19

**Authors:** Ingrid Egerod, Malene Brorsen Jensen, Suzanne Forsyth Herling, Karen-Lise Welling

**Affiliations:** 1 Copenhagen University Hospital, Rigshospitalet, Department 7331, UCSF, Blegdamsvej 9, 2100 Copenhagen O, Denmark; 2 Neurointensive unit 2093, Department of Neuroanaesthesiology, The Neuroscience Centre, Copenhagen University Hospital, Rigshospitalet, DK-2100 Copenhagen O, Denmark; 3 Orthopedic department T, Herlev Hospital, DK-2730 Herlev, Denmark

## Abstract

**Introduction:**

Sedation protocols are needed for neurointensive patients. The aim of this pilot study was to describe sedation practice at a neurointensive care unit and to assess the feasibility and efficacy of a new sedation protocol. The primary outcomes were a shift from sedation-based to analgesia-based sedation and improved pain management. The secondary outcomes were a reduction in unplanned extubations and duration of sedation.

**Methods:**

This was a two-phase (before-after), prospective controlled study at a university-affiliated, 14-bed neurointensive care unit in Denmark. The sample included patients requiring mechanical ventilation for at least 48 hours treated with continuous sedative and analgesic infusions or both. During the observation phase the participants (n = 106) were sedated as usual (non-protocolized), and during the intervention phase the participants (n = 109) were managed according to a new sedation protocol.

**Results:**

Our study showed a shift toward analgo-sedation, suggesting feasibility of the protocol. We found a significant reduction in the use of propofol (*P *< .001) and midazolam (*P *= .001) and an increase in fentanyl (*P *< .001) and remifentanil (*P *= .003). Patients selected for daily sedation interruption woke up faster, and estimates of pain free patients increased from 56.8% to 82.7% (*P *< .001), suggesting efficacy of the protocol. The duration of sedation and unplanned extubations were unchanged.

**Conclusions:**

Our pilot study showed feasibility and partial efficacy of our protocol. Some neurointensive patients might not benefit from protocolized practice. We recommend an interdisciplinary effort to target patients requiring less sedation, as issues of oversedation and inadequate pain management still need more attention.

**Trial registration:**

ISRCTN80999859.

## Introduction

Most mechanically ventilated patients in the intensive care unit (ICU) need sedation and analgesia to maintain comfort, relieve anxiety, facilitate care, and adapt to ventilatory support [1]. Although a variety of indications for sedation exist, recent years have seen a general trend toward lighter sedation for the mechanically ventilated patient [2]. Daily sedation interruption has been used as a way of reducing escalation of medication doses and a new study has demonstrated the feasibility of a protocol of no sedation [3]. Another trend has been the reversal from sedation-analgesia to analgo-sedation, with the primary goal of addressing pain and discomfort, and then if necessary, adding sedation [4-7]. Variations in the management of sedation, analgesia, and neuromuscular blockade have been demonstrated internationally [2,8-12]. Protocols have been introduced to increase consistency, and studies have shown that sedation protocols may decrease the duration of mechanical ventilation, the incidence of ventilator-associated pneumonia (VAP), and improve the probability of successful extubation in general ICUs [13-15].

Guidelines for the sustained use of sedatives and analgesics have been developed for patients in the general ICU [16], but little attention has been directed toward sedation of patients in the neurointensive care unit (NICU). Although neurointensive patients share many goals with general ICU patients, some indications are unique to the NICU population, such as maintaining adequate cerebral perfusion pressure (CPP), while controlling intracranial pressure (ICP) and mean arterial pressure (MAP) [17,18]. Sedation in NICU is complicated due to many specific, and sometimes conflicting, indications. Conditions such as delirium, anxiety and pain may all result in agitation, but need to be treated individually [19,20]. One particular challenge is the inability to adequately assess the sedation level, and to distinguish between the indications for sedation and for pain relief, due to the reduced level of consciousness. We suspected that over-sedation and inadequate pain management might be evident at our study site. The aim of the present study was to describe sedation practice at a neurointensive care unit and to assess the feasibility and efficacy of a sedation protocol based on the principles of analgo-sedation.

## Material and methods

### Design

**Figure 1:**
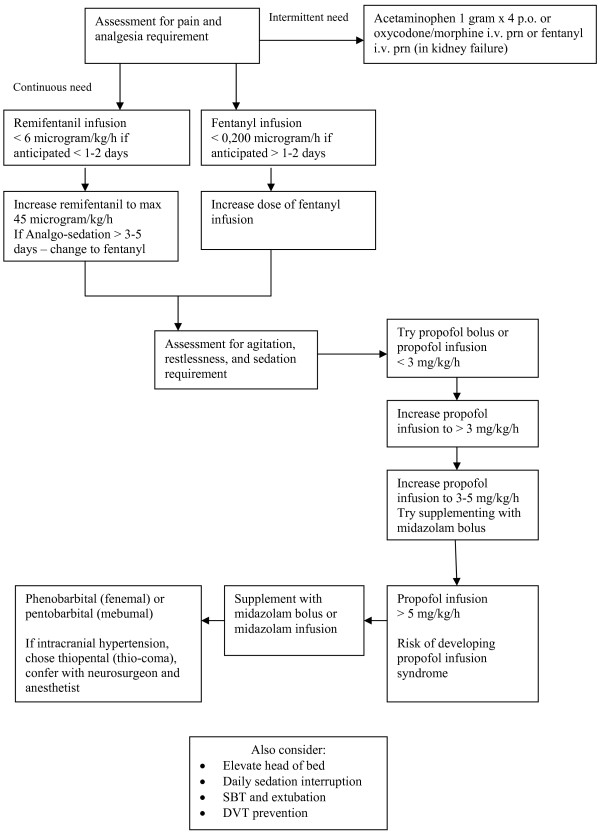
**Flow-diagram for analgo-sedation protocol**.

The following was an interventional non-randomized controlled single-center trial at a 14-bed NICU at a 1,082-bed university hospital in Denmark. The study consisted of an observational period of usual care, and an interventional period using a multidisciplinary evidence-based protocol for analgo-sedation. Usual care at our study site consisted of non-protocolized sedation and mechanical ventilation. We developed a 10-page sedation protocol for the study, which was implemented during an eight-month interim between the two study periods. The sedation protocol was introduced to the participants after the observational period to avoid contamination by prior knowledge of the protocol. The flow-diagram from our sedation protocol is shown in Figure 1. In order to obtain adherence to the study, two nurses were allocated to manage implementation of the sedation protocol between the study periods, and facilitate data collection throughout the study. The two nurses did not manage sedation practice during the intervention period.

The primary outcomes were (1) a shift from sedation-based to analgesia-based sedation (analgo-sedation), assessed from the changes in the use of sedatives and analgesic agents in each patient, and (2) improved pain management, assessed daily in each patient using the Pain Intensity scale [21]. The secondary outcomes were a reduction in unplanned extubations and duration of continuous sedation or analgesia. Unplanned extubations included deliberate (patient involvement) and accidental extubations.

### Participants

**Figure 2:**
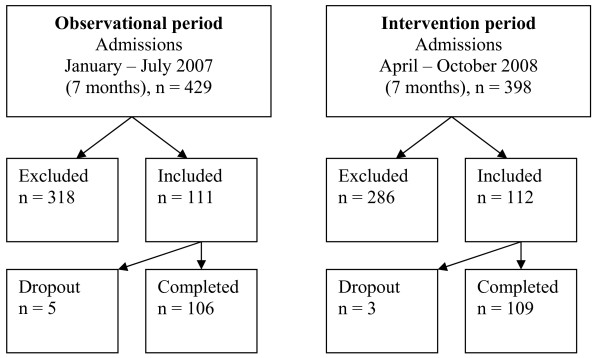
**Trial profile**.

Participants were consecutively enrolled in the study during two seven-month periods in January through July 2007 and April through October 2008; 106 participants were recruited for the first study period and 109 new participants were recruited for the second study period (215 participants recruited in total, Figure 2).

### Inclusion criteria

The inclusion criteria were as follows: male and female patients > 17 years of age admitted to NICU; patients mechanically ventilated ≥ 48 hours; and patients receiving continuous infusions of sedatives and analgesics or both.

### Exclusion criteria

Patients excluded from the study were those transferred intubated from other units to the NICU; patients receiving infusions of sedatives or analgesics < 48 hours; patients intubated later than 24 hours after NICU admission; and potential organ donors or patients receiving terminal care.

### Intervention

The sedation protocol was developed by an interdisciplinary team consisting of nurses and physicians at the study site, and the protocol was subsequently externally evaluated by experts at other hospitals, who were asked to assess and provide feed-back on the protocol. Conception of the protocol was inspired by the clinical practice guideline developed by the Society of Critical Care Medicine for general intensive care patients [16]. In addition to this, the protocol was written in accordance with the principles for analgo-sedation [4], and adjusted to meet the specific indications for sedation in neurointensive patients [22], for example, subarachnoid hematoma, traumatic brain injury, medullar injury, status epilepticus, and neuromuscular disease. The 10-page analgo-sedation protocol included the following general and specific recommendations, for example: Analgesics (fentanyl, remifentanil, oxycodone) should be given before a sedative; Sedatives (propofol or midazolam) should be administered only if necessary; Remifentanil should be administered for short-term sedation only (less than three to five days); Fentanyl (not remifentanil) should be administered for patients with traumatic brain injury.

### Data collection

Baseline characteristics at inclusion were sex, age, weight, APACHE II (Acute Physiology and Chronic Health Evaluation), SAPS II (Simplified Acute Physiology Score), SOFA (Sequential Organ Failure Assessment), and admission diagnosis. The study protocol included daily registration of medication doses (continuous infusions and boluses of propofol, midazolam, fentanyl, remifentanil, morphine, and oxycodone), sedation level (Ramsay Sedation Scale), pain level (Pain Intensity Scale), and level of consciousness (Glasgow Coma Scale, GCS). In addition, daily sedation interruption, duration of sedation interruption, late pneumonia, and accidental extubations were recorded. Sedation level was assessed by nurses once on each shift (day, evening, night) on the six-point Ramsay Sedation Scale (RSS): agitated/restless = 1, oriented/cooperative = 2, responding to commands only = 3, brisk response to light glabellar tap = 4, sluggish response to light glabellar tap = 5, no response = 6 [23]. The target sedation level was RSS 2 to 3 (in patients with increased ICP, the target was 5 to 6). Pain was estimated during each shift in conjunction with the sedation level on the six-point Pain Intensity scale (PI): no pain = 1, mild pain = 2, moderate pain = 3, severe pain = 4, very severe pain = 5, worst possible pain = 6 [21]. Pain estimation was determined by alterations in vital signs (blood pressure, heart rate, respiratory frequency) and facial expression (grimacing), and behavior (agitation). Interrater reliability was calculated prior to the study by 10 paired assessments (weighted kappa = 0.62, *P *= .04). The target pain level was PI score 1 to 2. GCS was assessed during daily sedation interruption, and during each shift along with the sedation new level. Daily sedation interruption was not routine for all patients, but had to be ordered by the physician on rounds. The sedation protocol reads: 'The physician on rounds determines whether a wake-up call is appropriate (...) Daily wake-up call should be avoided in patients with elevated ICP or other neurosurgical contraindications.'

### Statistical analysis

Comparisons between the observational and intervention group were performed using the Chi-Square test or Fisher's exact test for categorical variables, the Mann-Whitney test for non-parametric variables, and Student's *t*-test for comparing the means of normally distributed independent-samples. A *P *value of ≤ .05 was considered statistically significant. The data analysis was performed using SPSS (Statistical Package for the Social Sciences version 17, SPSS Inc., an IBM Company Headquarters, Chicago, Illinois).

### Ethical considerations

The study was conducted in accordance with Good Clinical Practice and the guidelines of the Declaration of Helsinki; it was acknowledged by the Danish Data Protection Agency (J.nr.2006-41-7419) and the Danish National Committee on Biomedical Research Ethics (J.nr.KF01-2006-4507). There was no requirement of informed consent because new drugs were not introduced.

## Results

A total of 827 patients were admitted during the inclusion periods, 604 were excluded, yielding 223 eligible patients. Eight patients dropped out, while 215 (94% of the eligible patients) completed the study (Figure 2). The baseline characteristics in the two groups were similar (Table 1). The main reasons for exclusion were intubation later than 24 hours after admission, terminal care, and organ donation. The reasons for dropout were intubation less than 48 hours, and organ donation.

**Table 1:** Baseline characteristics at inclusion

	Observational period (n = 106)	Intervention period (n = 109)	*P *Value
Male, n (%)	73 (69%)	65 (60%)	.159
Female, n (%)	33 (31%)	44 (40%)	.159
Age, mean years (SD)	55 (15)	52 (17)	.100
Weight, mean kg (SD)	78 (14)	75 (16)	.267
APACHE II, mean score (SD)	20 (4)	21 (5)	.477
SAPS II, mean score (SD)	51 (10)	50 (11)	.500
SOFA, mean score (SD)	8 (2)	8 (2)	.707
Diagnosis, n (%)			.186
SAH/ICH	50 (47%)	37 (34%)	
SDH/TBI	37 (35%)	41 (38%)	
Other	19 (18%)	30 (28%)	

Sex and diagnosis (Chi-Square test); age, weight, APACHE II, SAPS II, SOFA (t-test); and *P *value ≤ .05 significant.

ICH: intracerebral hemorrhage; SAH: subarachnoid hemorrhage; SDH: subdural hematoma; TBI: traumatic brain injury. Other: Medullar lesion, status epilepticus, neuromuscular disease, tumors, infections.

Hospital stay, NICU stay, duration of mechanical ventilation, duration of sedation, and unplanned extubation were unchanged. The relatively short hospital stay reflects patient transfer to other facilities offering neuro-rehabilitation. The small number of unplanned extubations does not permit evaluation with sufficient power. NICU mortality decreased non-significantly from 30% to 24% (Table 2).

**Table 2:** Length of stay, duration of treatment, complications, and mortality

	Observational period (n = 106)	Intervention period (n = 109)	*P *Value
Hospital stay, n (mean days) SD	12 (1 to 34) 7.9	12 (2 to 42) 8.8	.861
NICU stay, n (mean days) SD	9 (1 to 28) 6.6	9 (1 to 33) 7.1	.526
Mechanical ventilation, mean days (range) SD	6 (1 to 24) 5.3	7 (1 to 25) 6.2	.346
Sedation, mean days (range) SD	5 (1 to 19) 3.9	5 (1 to 18) 4.0	.575
Unplanned extubation, n (%)	7 (1%)	8 (3%)	
NICU mortality, n (%)	32 (30%)	25 (24%)	.353

Length of stay and duration of treatment (t-test) and unplanned extubation (Chi-Square test).

The sedation level was unchanged in the two study groups with a mean Ramsay score at 4.38/4.41 respectively (Table 3). The proportion, however, of RSS 2 to 3 (oriented and cooperative) increased from 10% to 19% and RSS 1 (agitated) decreased from 2% to 0.5% (*P *= .035). The mean GCS during daily sedation interruption was unchanged at 8.19/8.28 (Table 3), whereas the number of patients selected for daily awakening decreased from 47 (44%) to 22 (20%) among the patients awakened. Although the number of patients decreased, the frequency of daily awakening among these patients increased from 2.6 daily awakening trials to 6.7 (*P *= .003). The mean duration of sedation interruption (delay before GCS was assessed) decreased from 30 to 60 minutes to < 30 minutes (*P *= .001), with an increase from 48.0% to 69.1% of sessions lasting < 30 minutes (*P *= .001). The pain intensity score was significantly reduced from 1.54 in the observational period to 1.24 in the intervention period (*P *< .001).

**Table 3:** Level of sedation, consciousness, and pain (Ramsay, GCS, and PI)

	Observational period (n = 106)	Intervention period (n = 109)	*P *Value
Ramsay score mean (SD)	4.38 (1.21)	4.41 (1.25)	.651
GCS mean during sedation (SD)	7.49 (2.81)	7.42 (3.03)	.688
GCS mean during sedation interruption (SD)	8.19 (2.92)	8.28 (2.79)	.807
Duration of sedation interruption, n (%) - mean minutes delay	47 (44%) 30 to 60	22 (20%) <30	.001*
PI score mean (SD)	1.54 (.73)	1.24 (.61)	<.001*

Ramsay Sedation Score & Glasgow Coma Score, GCS (t-test); Pain Intensity Score, PI (Mann-Whitney test);

* *P *value ≤ .05 significant.

The distribution of pain-level assessments is shown in Table 4. The estimation of *no pain *increased from 56.8% to 82.7% of the recordings (*P *< .001).

**Table 4:** Distribution of pain-level scores (PI)

	Observational period		Intervention period	
	
PI-assessments	Count	%	Count	%
No pain	288	56.8	420	82.7
Mild pain	171	33.7	68	13.4
Moderate pain	44	8.7	12	2.4
Severe pain	2	0.4	5	1.0
Very severe pain	1	0.2	2	0.4
Worst possible pain	0	0.0	1	0.2
	507	100.0	508	100.0

PI, pain intensity

The medication pattern shifted from sedative to analgesic infusions. Table 5 shows a significant reduction in the daily dose of propofol and midazolam, and a significant increase in the daily dose of fentanyl and remifentanil. The number of days morphine was used decreased significantly, while the use of oxycodone increased in accordance with the protocol recommending a shift from morphine to oxycodone. The use of remifentanil increased as per protocol, but the agent was used beyond the three to five consecutive days recommended. During the observational period 18 cases lasted 6 to 16 days, and during the intervention period 22 cases lasted 6 to 19 days.

**Table 5:** Continuous sedative and analgesic infusions

	Observational period (n = 106, days = 553)	Intervention period (n = 109, days = 556)	*P *Value
Propofol days (% of total days)	307 (56%)	283 (51%)	.006*
Mean dose/day (mg) ± SD	2,592 ± 1,623	2,074 ± 1,308	< .001*
Range (mg)	550 to 7,910	510 to 7,070	
			
Midazolam days (% of total days)	49 (9%)	80 (14%)	.078
Mean dose/day (mg) ± SD	238 ± 152	157 ± 122	.001*
Range (mg)	3 to 485	0 to 425	
			
Fentanyl days (% of total days)	137 (25%)	114 (21%)	.171
Mean dose/day (mcg) ± SD	2,303 ± 1,606	4,919 ± 3,588	< .001*
Range (mcg)	80 to 8,000	100 to 16,000	
Remifentanil days (% of total days)	297 (54%)	420 (76%)	.281
			
Mean dose/day (mcg) ± SD	6,888 ± 5,373	8,233 ± 6,384	.003*
Range (mcg)	1,000 to 18,000	1,000 to 19,000	
			
Morphine days (% of total days)	41 (7%)	7 (1%)	.009*
Mean dose/day (mg) ± SD	16 ± 9	10 ± 0	.077
Range (mg)	7 to 40	10 to 10	
			
Oxycodone days (% of total days)	67 (12%)	74 (13%)	.199
Mean dose/day (mg) ± SD	25 ± 22	20 ± 14	.079
Range (mg)	5 to 100	3 to 69	

Number of days (t-test), medication dose (Mann-Whitney), and * *P *value ≤ .05 significant. Range was calculated within limits of adequate sedation.

## Discussion

The aim of the study was to describe sedation practice at a neurointensive care unit and to assess the feasibility and efficacy of a sedation protocol based on the principles of analgo-sedation. Current sedation practice at a Danish NICU in 2007 (observation period) was non-protocolized sedation and mechanical ventilation, using primarily continuous infusions of propofol and midazolam for sedation and fentanyl and morphine for analgesia. This is consistent with findings from general ICUs in Denmark in 2003 [2]. The primary outcomes were a shift from sedation-based to analgesia-based sedation, and improved pain management. Our study showed a significant reduction in the use of propofol and midazolam and an increase in the use of fentanyl and remifentanil, suggesting feasibility of our protocol. The patients selected for daily sedation interruption woke up faster, and more patients were estimated to be pain free, supporting our suspicion and suggesting efficacy of our protocol. The secondary outcomes of fewer unplanned extubations and shorter duration of sedation were unchanged, suggesting limited efficacy of our protocol.

The sedation level was measured by the Ramsay Sedation Scale, which was chosen for pragmatic reasons. It is the most commonly used sedation scoring system in Denmark, where the scale is integrated with the computerized Patient Data Management system at many hospitals [2]. The Ramsay scale does not require patient cooperation and is used at about 40% of Danish ICUs, as compared to 38% in a study of North American hospitals [2,24]. Other sedation scoring systems used in Denmark are the Motor Activity Assessment Scale (MAAS) 15%, the Riker Sedation-Agitation Scale (SAS) 12%, the Richmond Agitation Sedation Scale (RASS) 9%, and the Cook and Palma Scale (COOK) 7% [25]. Many of these sedation scales have demonstrated validity and good inter-rater reliability [26].

The mean sedation level in our study was Ramsay 4 to 5 (brisk to sluggish response), which is comparable to the mean SAS score of 3 to 4 (difficult to rouse) found in similar studies of neurointensive patients [21]. Although the proportion of assessments at the target level of Ramsay 2 to 3 increased, it was unrealistic to reach this level in all patients. One reason is that many patients were unresponsive due to their neurologic condition or drug accumulation. Another reason is that the protocol recommended Ramsay 5 to 6 in patients with elevated ICP. These issues demonstrate some of the challenges of sedation management in neurointensive patients [27].

Many patients were sedated with remifentanil, which should permit rapid awakening in the neurologically unimpaired. Due to the ultra-short duration of action, the protocol did not recommend bolus remifentanil [21]. Also, due to lack of clinical evidence, the protocol did not recommend using remifentanil more than five days consecutively. Although the similar baseline characteristics, length of stay, and sedation scores in the before-after study suggest that data were recorded accurately, the extended continuous use of remifentanil shows that adherence to the protocol was inadequate. More studies are needed to determine the consequences of long-term use of remifentanil.

The duration of mechanical ventilation remained unchanged in the two groups in our study. Studies of general ICU patients have demonstrated significantly shorter median duration of mechanical ventilation using a nurse-implemented sedation protocol [13,15,28]. Our findings may, in part, be due to the consistently low level of consciousness in our population. A GCS of 3 to 8 has been used as an indication for intubation in trauma patients [29]. The small number of unplanned extubations may be attributed to over-sedation or reduced level of consciousness. The NICU mortality and number of unplanned extubations in our study are comparable to general ICUs, as were the APACHE II and SAPS II scores [15,28].

Fewer patients in the intervention period were *slow to awake*, which might be ascribed to decreased use of midazolam (long-acting sedative) and less drug accumulation. Although the use of fentanyl increased, the problem of drug accumulation is less severe than in midazolam [30]. The shift from fentanyl to remifentanil (short acting opiate) also led to a shorter delay in awakening. The shift to analgo-sedation might explain why fewer patients were estimated to be in pain. Fewer patients were selected for daily awakening, but the frequency increased, suggesting that the selection of patients was more appropriate in the intervention period, and was a sign of adherence to the protocol. Sedation was not interrupted in patients that required deeper sedation, but among patients requiring less sedation, fewer were over-sedated, even prior to awakening.

Our study had several limitations. This was a single-center study, which means that the results may not translate to other units. Due to the small population of the country, the volume of neurointensive patients is limited, but a multicenter study would have been a stronger design. The choice of a before-after design was not optimal, but a randomized study was not feasible at the unit due to risk of control group contamination. The Pain Intensity scale is inherently subjective as it depends on the estimate of the clinician. In the future, a more accurate scoring system for pain in the unresponsive patient needs to be developed and validated. The similar GCS in the observational and intervention periods in the study increased the internal validity. Also, the mean GCS was similar to other studies, thus increasing the external validity of our findings [19]. The study design would have been stronger if intracranial pressure, cerebral perfusion pressure and mean arterial pressure had been recorded on relevant patients. Finally, the extended use of remifentanil suggests that the period of implementation should have been longer, ensuring better adherence to the protocol. We have seen a positive effect of the protocol on management of sedation and pain, but the sample is small, and more attention needs to be directed towards sedation issues in the NICU population in general and the needs of sub-groups of patients in particular.

## Conclusions

This pilot study showed feasibility and partial efficacy of our protocol. Some neurointensive patients might not benefit from protocolized practice because the duration of mechanical ventilation is more influenced by level of consciousness related to underlying illness, and the ongoing need for airway protection, and less influences by sedatives and analgesics. We recommend an interdisciplinary effort to target patients requiring less sedation, as issues of over-sedation and inadequate pain management in neurointensive patients still need more attention.

## Key messages

• Sedation protocols for neurointensive patients are needed

• Neurointensive patients are difficult to assess for degree of pain and sedation due to reduced level of consciousness

• Validated instruments for assessment of pain and sedation levels in patients unable to self-report are needed

• The presented analgo-sedation protocol resulted in more pain free patients and in shorter delays in daily awakening of selected patients

## Abbreviations

APACHE II: acute physiology and chronic health evaluation; COOK: Cook and Palma scale; CPP: cerebral perfusion pressure; GCS: Glasgow coma scale; ICH: intracerebral hemorrhage; ICP: intracranial pressure; ICU: intensive care unit; MAAS: motor activity assessment scale; MAP: mean arterial pressure; NICU: neurointensive care unit; PI: pain intensity scale; RASS: Richmond agitation sedation scale; RSS: Ramsay sedation scale; SAH: subarachnoid hemorrhage; SAPS II: simplified acute physiology score; SAS: Riker sedation-agitation scale; SD: standard deviation; SDH: subdural hematoma; SOFA: sequential organ failure assessment; SPSS: statistical package for the social sciences; TBI: traumatic brain injury; VAP: ventilator-associated pneumonia.

## Competing interests

The authors declare that they have no competing interests.

## Authors' contributions

All authors contributed to the conception and design of the study and revision and final approval of the manuscript. IE conducted analysis and interpretation and wrote the first draft of the manuscript. MBJ and SFH managed data acquisition, and KLW wrote the first draft of the sedation protocol.
